# Modulation of arterial intima stiffness by disturbed blood flow

**DOI:** 10.3389/ebm.2024.10090

**Published:** 2024-07-31

**Authors:** Briana C. Bywaters, Andreea Trache, Gonzalo M. Rivera

**Affiliations:** ^1^ Department of Veterinary Pathobiology, Texas A&M University, College Station, TX, United States; ^2^ Department of Medical Physiology, Texas A&M Health Science Center, Bryan, TX, United States; ^3^ Department of Biomedical Engineering, Texas A&M University, College Station, TX, United States

**Keywords:** arterial stiffness, disturbed blood flow, endothelium, subendothelial matrix, atomic force microscopy, lysyl oxidase, collagen crosslinking, BAPN

## Abstract

The intima, comprising the endothelium and the subendothelial matrix, plays a crucial role in atherosclerosis pathogenesis. The mechanical stress arising from disturbed blood flow (d-flow) and the stiffening of the arterial wall contributes to endothelial dysfunction. However, the specific impacts of these physical forces on the mechanical environment of the intima remain undetermined. Here, we investigated whether inhibiting collagen crosslinking could ameliorate the detrimental effects of persistent d-flow on the mechanical properties of the intima. Partial ligation of the left carotid artery (LCA) was performed in C57BL/6J mice, inducing d-flow. The right carotid artery (RCA) served as an internal control. Carotids were collected 2 days and 2 weeks after surgery to study acute and chronic effects of d-flow on the mechanical phenotype of the intima. The chronic effects of d-flow were decoupled from the ensuing arterial wall stiffening by administration of β-aminopropionitrile (BAPN), an inhibitor of collagen crosslinking by lysyl oxidase (LOX) enzymes. Atomic force microscopy (AFM) was used to determine stiffness of the endothelium and the denuded subendothelial matrix in *en face* carotid preparations. The stiffness of human aortic endothelial cells (HAEC) cultured on soft and stiff hydrogels was also determined. Acute exposure to d-flow caused a slight decrease in endothelial stiffness in male mice but had no effect on the stiffness of the subendothelial matrix in either sex. Regardless of sex, the intact endothelium was softer than the subendothelial matrix. In contrast, exposure to chronic d-flow led to a substantial increase in the endothelial and subendothelial stiffness in both sexes. The effects of chronic d-flow were largely prevented by concurrent BAPN administration. In addition, HAEC displayed reduced stiffness when cultured on soft vs. stiff hydrogels. We conclude that chronic d-flow results in marked stiffening of the arterial intima, which can be effectively prevented by inhibition of collagen crosslinking.

## Impact statement

Although influenced by systemic risk factors, atherosclerotic plaques develop in response to mechanical forces impacting bifurcations and curved segments of the arterial tree. Unraveling the intertwined effects of disturbed blood flow and arterial wall stiffening in the pathogenesis of atherosclerosis remains a challenge. This study provides novel insights into pathological vascular remodeling, emphasizing the significant impact of chronic d-flow on arterial intima stiffening. Our research demonstrates that inhibiting collagen crosslinking effectively prevents the substantial stiffening of the arterial intima induced by chronic exposure to d-flow. Accordingly, modulation of intimal stiffness emerges as a promising avenue for the development of innovative targeted interventions aimed at promoting arterial health.

## Introduction

Atherosclerotic lesions develop primarily in areas where arteries branch out or have curved segments [1], despite the influence of systemic risk factors [2]. The mechanical forces exerted by local blood flow patterns play a crucial role in determining the endothelial phenotype and influencing the progression of atherosclerosis [3, 4]. In straight arterial regions, the high-magnitude and unidirectional shear stress resulting from steady blood flow promotes endothelial health and is considered protective against atherosclerosis. On the other hand, in bifurcations and curved arterial segments, the low-magnitude and oscillatory shear stress caused by disturbed blood flow (d-flow) leads to endothelial dysfunction and is deemed prone to atherosclerosis.

The mechanical properties of the arterial wall also play a significant role in the development and progression of atherosclerosis [5]. Factors such as aging [6], metabolic imbalance [7], and hypertension [8], contribute to arterial stiffening by causing changes in the extracellular matrix and vascular cells. Arterial stiffening is also observed in bifurcations and curved segments, which aligns with the localized formation of atherosclerotic lesions due to d-flow [9–11]. However, the specific impact and interplay between chronic exposure to d-flow and arterial wall stiffening on the intimal phenotype, and specifically its biomechanical properties, remain poorly understood.

The mouse model of partial carotid ligation (PCL) has been valuable in uncovering the mechanisms involved in the development of atherosclerosis [12–14]. By surgically inducing d-flow in a previously healthy and straight arterial segment, the development of atherosclerotic lesions can be rapidly stimulated in hyperlipidemic mice [12, 15, 16]. In the presence of physiological levels of cholesterol and triglycerides, however, prolonged exposure (∼2 weeks) to PCL-induced d-flow leads to arterial wall remodeling and stiffening, similar to the effects of aging [17]. This model offers, therefore, a unique opportunity to study how mechanical forces contribute to abnormal molecular patterns and cellular phenotypes that drive the progression of atherosclerosis [14]. The objective of this research was to determine the specific contribution of d-flow and arterial wall stiffening to the mechanical phenotype of components of the arterial intima, i.e., the intact endothelium and the denuded subendothelial matrix. This study tested the hypothesis that chronic d-flow initiates a feed forward loop promoting arterial wall stiffening leading to altered endothelial mechanics; hence interventions aimed at reducing arterial wall stiffness have the potential to disrupt this loop and preserve the mechanical homeostasis of the endothelium.

Our findings indicate that reducing arterial wall stiffness can reverse the detrimental effects of prolonged exposure to d-flow on the biomechanical properties of the arterial endothelium.

## Materials and methods

### Animals

Mice were maintained in the Comparative Medicine Program Laboratory Animal Resources and Research facility at Texas A&M University. Animal handling and experimental procedures were performed according to a protocol (AUP 2019-0184) approved by the Institutional Animal Care and Use Committee of Texas A&M University. Eight-week old male and female C57BL/6J mice (RRID:IMSR_JAX:000664) were used for determinations of stiffness using atomic force microscopy (AFM). The fluorescence reporter mT/mG mouse [18], expressing tamoxifen-inducible Cre recombinase (*Cdh5*-Cre^ERT2^) in the endothelium [19], was used to verify arterial wall integrity and the subsequent mechanical removal of the endothelium. In these mice, endothelial cells were labeled with EGFP following five intraperitoneal injections, administered every other day, of 75 mg/kg tamoxifen (Sigma-Aldrich) in 100 μL corn oil. All animals were maintained on a 12-h light/dark cycle and fed regular chow ad-libitum.

### Experimental design

**FIGURE 1:**
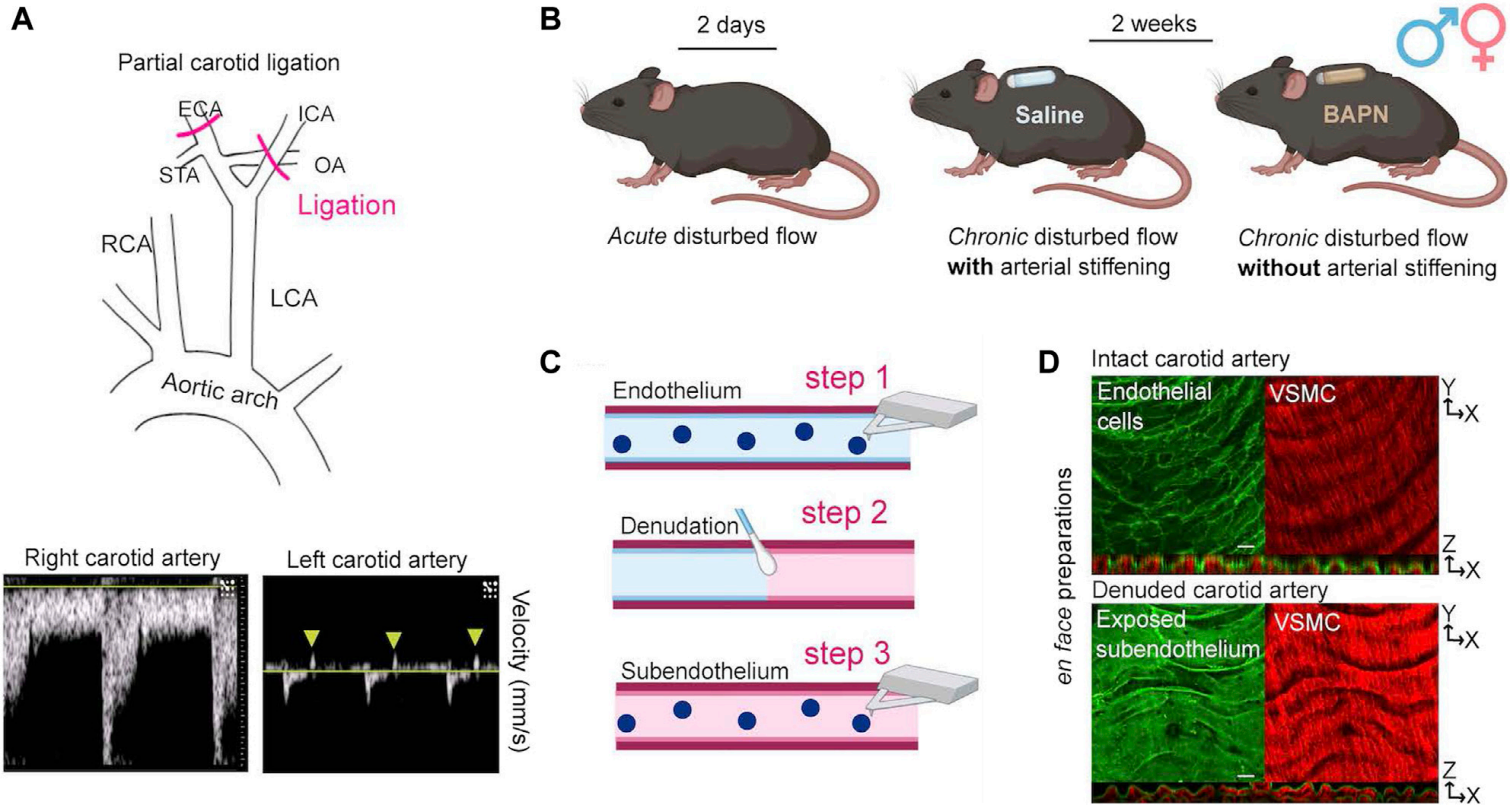
Experimental strategy for force decoupling and assessment of the arterial mechanical phenotype. **(A)** At 8 weeks of age, male and female C57B/6J mice underwent PCL of the left carotid artery (LCA) to induce disturbed hemodynamics (top). The intact right carotid artery (RCA) served as an internal control. Successful ligation was verified by ultrasound detection of both decreased blood flow velocity and blood flow reversal (arrowheads) in the ligated LCA (bottom). **(B)** Acute and chronic effects of disturbed hemodynamics were determined 2 days or 2 weeks after PCL (n = 6/sex/time). To decouple the effects of chronic (2 weeks) disturbed hemodynamics from the ensuing stiffening of the arterial wall, mice were affixed at the time of surgery with subcutaneous osmotic minipumps delivering either Saline or 150 mg/kg/day of BAPN, an inhibitor of the collagen crosslinking enzymes of the lysyl oxidase (LOX) family. **(C)** Both carotids were excised and opened *en face* for determination of the elastic modulus of intimal components by AFM. First, AFM measurements were collected from the intact endothelium (step 1). Subsequently, endothelial denudation was performed using a cotton swab (step 2). Following endothelial denudation, AFM measurements were collected from the exposed subendothelial matrix (step 3). **(D)** Successful handling of excised vessels was validated using an endothelial-specific fluorescence reporter mouse (Cdh5-Cre^ERT2^/mTmG) to confirm integrity (top) and removal (bottom) of the endothelium before and after denudation, respectively. Note apical endothelial fluorescence (top panel) and autofluorescence of elastic fibers (bottom panel) in orthogonal views. Scale bar is 20 μm. VSMC, vascular smooth muscle cells. ICA, internal carotid artery; OA, occipital artery; ECA, external carotid artery; STA, superior thyroid artery.

We performed partial ligation of the left carotid artery (Figure 1A) to determine the acute and chronic effects of d-flow on the mechanical phenotype of the endothelium and subendothelial matrix. Animals were sacrificed and carotid arteries collected 2 days or 2 weeks after surgery. The 2-day time point, which aligns with established experimental design principles in the field [14], captures the initial response to an acute change in hemodynamic forces [20]. To decouple the effects of chronic (2-weeks) d-flow from the ensuing arterial wall stiffening, animals were affixed with subcutaneous osmotic minipumps (ALZET model 1002, capacity: 100 μL, flow rate: 0.25 μL/h) delivering either phosphate buffered saline (Saline) or 150 mg/kg/day of β-aminopropionitrile (BAPN) [21], an inhibitor of the LOX family of enzymes (Figure 1B) [6, 17, 22–24].

### Surgical procedures

#### Partial carotid ligation (PCL)

Mice were anesthetized with isoflurane and hair was chemically removed (Nair, Ewing, NJ) from the chin to the bottom of the sternum. The exposed skin was swabbed alternately with iodine and isopropyl alcohol prior to incision. Sutures (0–6 silk) were used to occlude the external carotid, internal carotid, and occipital branches of the left carotid artery (LCA), leaving the superior thyroid artery unobstructed (Figure 1A). The right carotid artery (RCA) was left intact and served as an internal control. The incision was closed using tissue adhesive (Vetbond, 3M, Saint Paul, MN). Soon after surgery, all mice received a subcutaneous injection of buprenorphine (0.1 mg/kg) to minimize pain and discomfort.

#### Osmotic minipump insertion

Mice used for the 2-week experiments remained anesthetized after completion of the PCL procedure and were placed in a prone position. Hair was chemically removed from between the shoulder blades and the skin was swabbed with iodine and isopropyl alcohol. A small incision was made to allow the subcutaneous insertion of an osmotic minipump. Following the subcutaneous insertion of the osmotic minipump, the incision was closed using 7 mm wound clips (EZ Clip, Stoelting, Chicago, IL).

### Doppler ultrasound

We used ultrasound to confirm successful PCL 1 day after the surgical procedure. Mice were anesthetized using isoflurane and subjected to Doppler ultrasound using a Vevo 3100 system (FUJIFILM VisualSonics, Inc., Bothell, WA) in B-mode. Both flow reversal and decreased flow velocity were recorded in the ligated vessels [12, 13]. The presence of normal flow was confirmed in the intact vessels (Figure 1A, bottom panels).

### Tissue collection and handling

Two days or 2 weeks following PCL, mice were euthanized using CO_2_ asphyxiation followed by cervical dislocation. The vascular tree was perfused through the apex of the heart with ice-cold Dulbecco’s phosphate buffered saline (DPBS, Gibco, Waltham, MA) prior to excision of carotid arteries. Excised vessels were immediately opened *en face* using vannas scissors (Fine Science Tools, Vancouver BC, Canada)*,* affixed to 60 mm plastic dishes (BD Falcon, Franklin Lakes, NJ), and submerged in room temperature DPBS. *En face* vessel preparations were used to determine the stiffness of the intact endothelium and the subendothelial matrix [25]. After measurement of the intact endothelial surface was completed, the endothelial layer was mechanically removed using 10 swipes with a cotton swab (Figure 1C), DPBS was replaced, and measurements of the exposed subendothelial matrix were immediately collected. The integrity of the endothelium and exposure of the subendothelial matrix before and after mechanical removal of the endothelium, respectively, were confirmed by microscopic imaging of arteries harvested from the fluorescence reporter mT/mG mouse (Figure 1D).

### Culture, immunofluorescence staining, and imaging of endothelial cells

Human aortic endothelial cells (HAECs; ATCC, Manassas, VA) were cultured in EGM-2 culture media (Lonza Walkersville, Basel, Switzerland) on 0.1% gelatin-coated plastic dishes until ∼80% confluence. Cells were then seeded (passage 5) on soft (4 kPa) or stiff (50 kPa) polyacrylamide hydrogels coated with 0.2% collagen I (Matrigen, San Diego, CA) at a density of 40 × 10^4^/18 mm^2^ and cultured for 24 h. HAECs were fixed with 4% paraformaldehyde for 15 min at 37°C in phosphate-buffered saline (PBS). Fixed cells were permeabilized with 0.1% Triton X-100 and nonspecific binding was blocked using 3% bovine serum albumin (BSA) in PBS. Coverslips were incubated with primary mouse monoclonal anti-paxillin antibody (BD Biosciences, 1:1000) in blocking solution for 1 hour at room temperature, followed by Alexa Fluor 488 goat anti-mouse secondary antibody (Life Technologies, 1:500) for 1 hour at room temperature. To visualize actin filaments, cells were stained with Alexa Fluor 647 phalloidin (Life Technologies, 1:200). Images were acquired on an Olympus FV3000 confocal microscope using a PlanApoN SC2 60 × 1.4NA objective at 0.414 µm/px and Z-step of 0.31 µm. Z-stack images were projected as sum intensity. A cell area mask was created using Auto threshold with Huang method of the actin channel in ImageJ/FIJI [26]. Fluorescence intensity inside the mask was measured for actin and paxillin to determine mean intensity.

### Atomic force microscopy

A numerical code, unknown to the experimenter, was used to deidentify samples and treatments. To determine the stiffness of the intact endothelium and subendothelial matrix, atomic force microscopy (AFM) measurements were performed as previously described [25]. The AFM probes used were unsharpened silicon nitride cantilevers with a spring constant of 12.2 ± 0.4 pN/nm (MLCT Bio, Bruker Nano-Surfaces, Billerica, MA). The AFM was operated in force mode, setting the cantilever to touch and retract from the tissue surface at 0.8 μm/s in the z-axis. An average of 4–6 separate sites were measured on the common carotid artery along the segment between the aortic arch (proximal) and the internal/external carotid artery bifurcation (distal) with approximately 60 force curves acquired at each site. Care was taken to avoid obtaining measurements at the edges of the tissue due to eventual damage during handling and immobilization in the dish.

In the case of HAEC cultured on hydrogels, measurements of cells stiffness and integrin ⍺5β1 adhesion force to fibronectin were conducted using AFM tips functionalized with fibronectin [27, 28]. Individual cells were measured midway between the nucleus and the edge of the cell [29]. A minimum of 10 cells were measured in each independent experiment, for a total of 1330–1344 force curves per condition. Experiments were performed in triplicates.

### AFM data processing

Stiffness at the point of contact was evaluated as Young’s modulus of elasticity (E), by fitting the approach curve between the initial point of tissue contact and point of maximum probe displacement with Sneddon’s modified Hertz model [30]. Kernel density plots of the distribution of stiffness at the point of contact for both *ex vivo* vessels and cultured cells, as well as kernel density plots of the distribution of integrin adhesion force measurements for cultured cells, were generated using NForceR software [31].

### Second harmonic generation imaging

Vessels were mounted on glass slides prior to imaging. Collagen was visualized by second harmonic generation (SHG) using an Olympus Fluoview FVMPE-RS Multiphoton Laser Scanning Microscope equipped with a XLPLN25XWMP2 25x water immersion objective and 1045 nm excitation laser. Images were processed using in ImageJ/FIJI [26]. All images were subjected to a 50 pixel rolling ball background subtraction and maximum intensity projections of z-stacks were generated.

### Statistical analysis

We followed previously established guidelines for determining sample size in experiments involving laboratory animals [32]. Using data from preliminary studies, we estimated standardized effect sizes of 2.09 and 2.46 (stiffness, kPa), respectively, for males and females. To estimate the number of biological replicates, power analysis using G*Power (version 3.1.9.7.0) [33] was performed. Thus, it was determined that a sample size of n = 6 would have a 90% power to detect an effect size ≥2.09 assuming a 5% significance level and a two-sided test.

**FIGURE 2:**
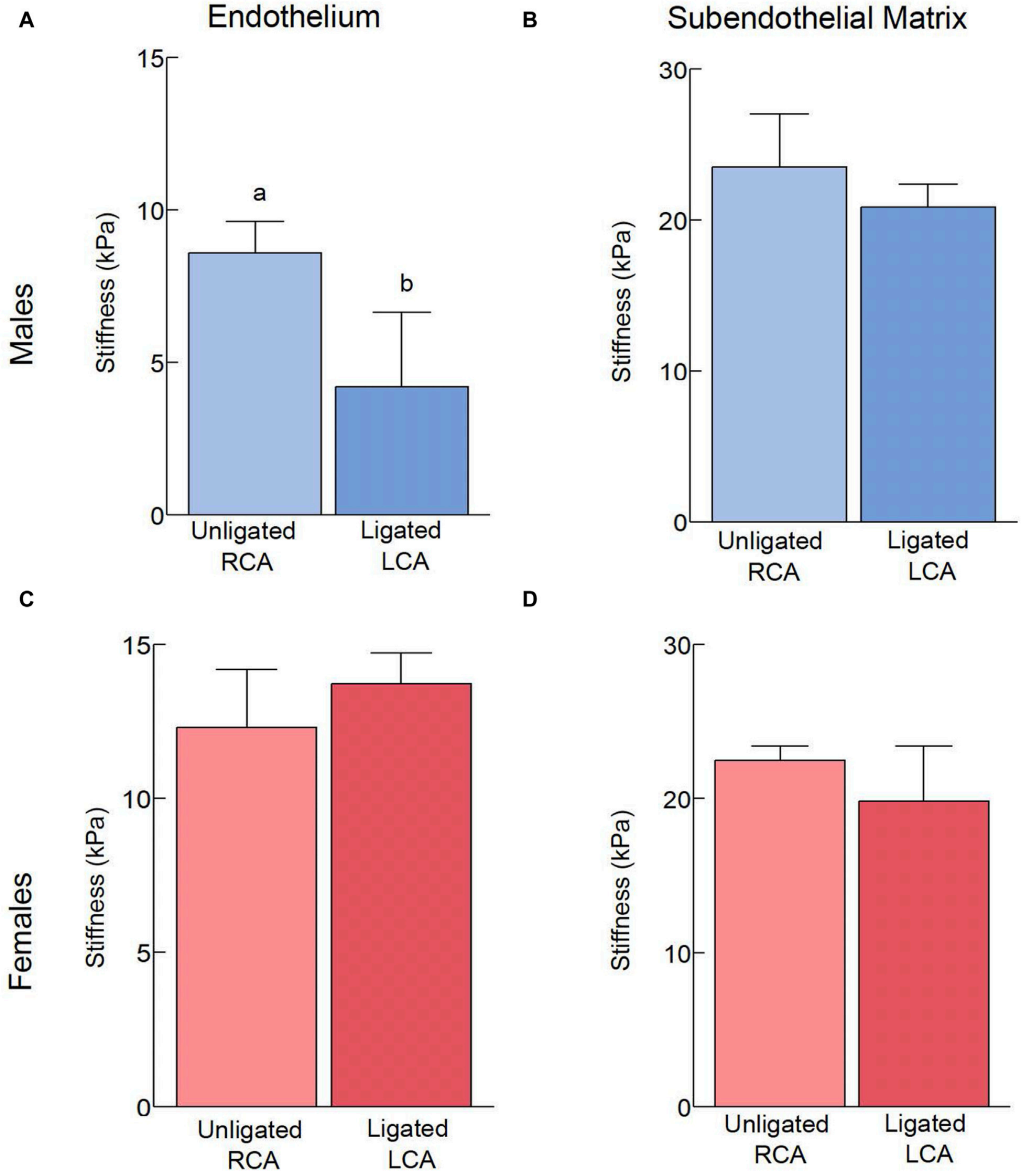
Effects of acute, 2-day exposure to disturbed blood flow on the mechanical properties of the carotid intima. The elastic modulus of the endothelium **(A,C)** and subendothelial matrix **(B,D)** was measured using AFM in male **(A,B)** and female **(C,D)** mice 2 days after PCL (n = 6/sex). The number of force curves acquired per vessel type is indicated in parentheses as follows: **(A)** Unligated RCA (1451), Ligated LCA (2609). **(B)** Unligated RCA (890), Ligated LCA (1370). **(C)** Unligated RCA (1103), Ligated LCA (1295). **(D)** Unligated RCA (852), Ligated LCA (902). Bars with different letters, *p* < 0.05.

**FIGURE 3:**
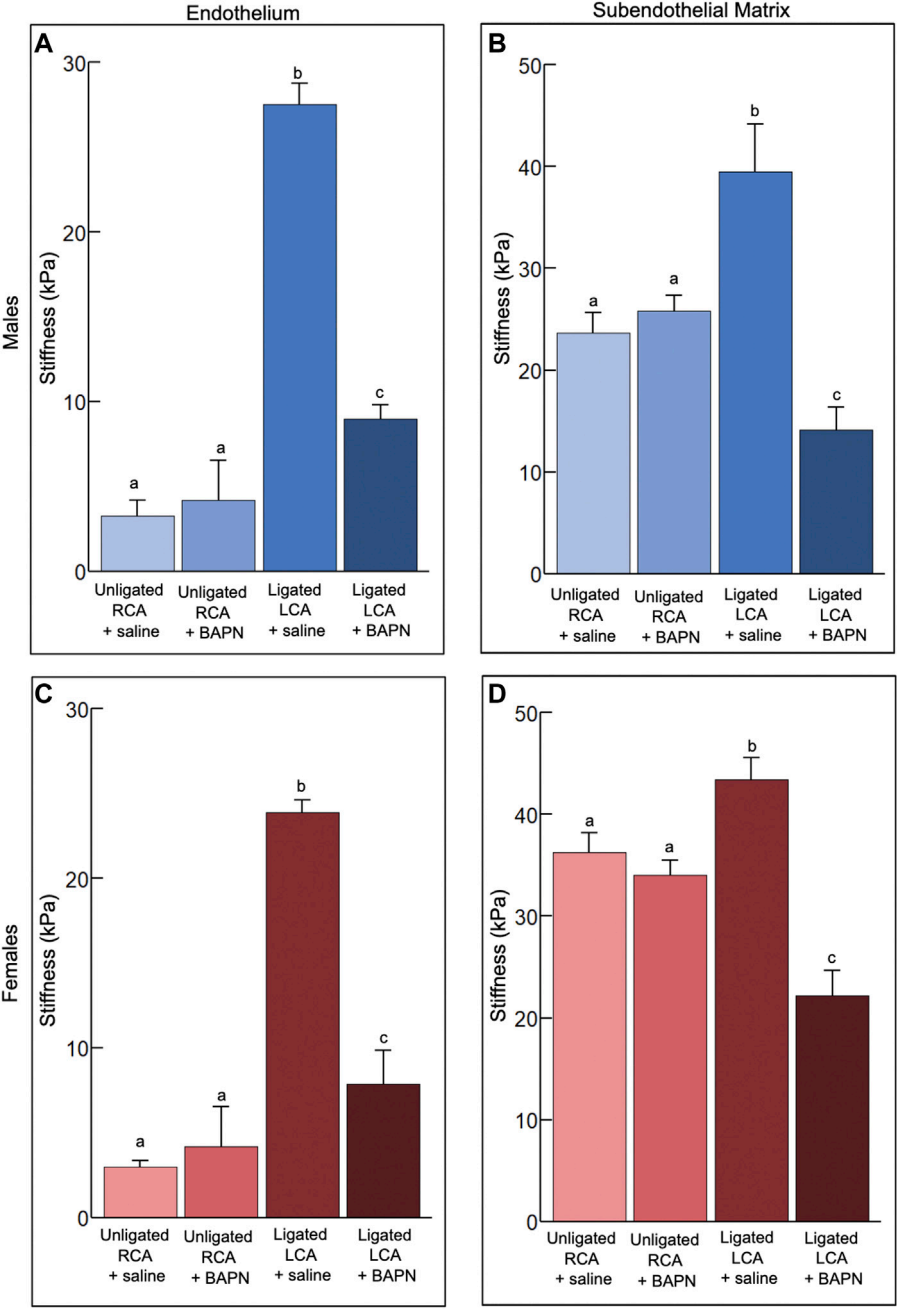
Effects of chronic, 2-week exposure to disturbed blood flow on the mechanical properties of the carotid intima. The elastic modulus of the endothelium **(A,C)** and subendothelial matrix **(B,D)** was measured using AFM in male **(A,B)** and female **(C,D)** mice 2 weeks after PCL and treatment with either Saline or 150 mg/kg/day of BAPN, an inhibitor of the collagen crosslinking enzymes of the LOX family (n = 6/sex/group). The number of force curves acquired per vessel type is indicated in parentheses as follows: **(A)** Unligated RCA + saline (1350), Unligated RCA + BAPN (3517), Ligated LCA + saline (2273), Ligated LCA + BAPN (2664). **(B)** Unligated RCA + saline (1995), Unligated RCA + BAPN (1007), Ligated LCA + saline (2251), Ligated LCA + BAPN (1716). **(C)** Unligated RCA + saline (831), Unligated RCA + BAPN (1675), Ligated LCA + saline (1592), Ligated LCA + BAPN (1421). **(D)** Unligated RCA + saline (1570), Unligated RCA + BAPN (1542), Ligated LCA + saline (1817), Ligated LCA + BAPN (890). Bars with different letters, *p* < 0.05.

We analyzed stiffness distribution at the point of contact in blood vessels using kernel density plots and Lorentzian fitting using Fityk software [34]. We generated 95% confidence intervals for peak stiffness values (see Figures 2, 3) for each vessel type [29, 35]. Statistically significant differences were identified based on non-overlapping confidence intervals (*p* < 0.05). Additionally, we introduced a stiffness variability index calculated from half-width half-max (HWHM) values of each fitted distribution. This index represents half the distance between the two points on the x-axis where the probability density function of the distribution drops to half of its maximum value. The stiffness variability index quantifies the spread of elastic modulus measurements for a given vessel type, combining data from approximately 60 force curves per site across 4–6 sites per sample and six independent biological replicates. Thus, a higher numerical value of the stiffness variability index corresponds to a broader range of elastic modulus measurements.

## Results

### Effects of acute exposure to d-flow on the mechanical properties of the carotid intima

To determine how acute d-flow affects the mechanical properties of the carotid intima, we harvested carotid arteries from male and female mice (n = 6/sex) 2 days following PCL. We used AFM to measure the stiffness of the intact endothelium and subendothelial matrix in both the intact RCA and ligated LCA. Acute exposure to d-flow in males slightly decreased (*p* < 0.05) the endothelial stiffness (Figure 2A) but had no effect on the stiffness of the subendothelial matrix (Figure 2B). Acute exposure to d-flow in females, on the other hand, did not affect (*p* > 0.05) the endothelial (Figure 2C) or subendothelial (Figure 2D) stiffness. Regardless of sex, the intact endothelium was softer (*p* < 0.05) than the subendothelial matrix.

To assess the spread of elastic modulus measurements for a given vessel type, the stiffness variability index was compared (Table 1). In males, the ligation induced a decrease in endothelial stiffness variability but no changes were observed in the subendothelial matrix. In females, the stiffness variability index remained unchanged and increased ∼3 fold, respectively, in the endothelium and the subendothelial matrix.

**TABLE 1:** Stiffness variability index in intact vessels (Unligated RCA) or vessels exposed to acute (2 days) d-flow post-ligation (ligated LCA).

		Unligated RCA	Ligated LCA
Endothelium	Males	23.9	11.1
	Females	22.3	20.6
Subendothelium	Males	33.7	32.4
	Females	13.1	40.1

In conclusion, acute exposure to d-flow slightly reduces endothelial stiffness in males but not in females, with the endothelium being softer than the subendothelial matrix regardless of sex.

### Effects of chronic exposure to d-flow on the mechanical properties of the carotid intima

How chronic (2 weeks) d-flow affects the mechanical properties of the carotid intima in male and female mice treated with saline or BAPN (n = 6/sex/condition) was also determined. Treatment with BAPN was implemented to decouple the effects of chronic d-flow from the ensuing stiffening of the arterial wall.

Treatment with BAPN did not affect (*p >* 0.05) the endothelial stiffness in unligated arteries. In saline-treated mice, regardless of sex, chronic d-flow induced a ∼8-fold increase (*p* < 0.05) in the stiffness of the endothelium (Figures 3A, C). Such response was reduced largely by the administration of BAPN (*p* < 0.05). Although BAPN effectively prevented ligation-induced endothelial stiffening, the endothelial stiffness of ligated LCA + BAPN carotids was ∼2 fold higher than that of unligated RCA counterparts. Treatment with BAPN increased the stiffness variability index in the endothelium of unligated arteries (Table 2).

**TABLE 2:** Stiffness variability index in intact carotids (Unligated RCA) and carotids exposed to chronic (2 weeks) d-flow (Ligated LCA) in the absence (saline) or presence of the LOX family inhibitor β-aminopropionitrile (BAPN).

		Unligated RCA + saline	Unligated RCA + BAPN	Ligated LCA + saline	Ligated LCA + BAPN
Endothelium	Males	5.1	21.4	24.9	13.3
	Females	2.1	15.7	19.0	19.3
Subendothelium	Males	34.7	18.4	64.8	19.8
	Females	30.1	41.9	56.9	38.1

Treatment with BAPN did not affect (*p* > 0.05) the subendothelial stiffness in unligated arteries. Chronic exposure to d-flow stiffened the subendothelial matrix in both sexes, an effect that was fully counteracted (*p* < 0.05) by concurrent BAPN treatment (Figures 3B, D). Consistent with these results, second harmonic generation (SHG) imaging shows more prominent collagen fibrils in the wall of LCA + saline compared to LCA + BAPN or unligated RCA arteries (Supplementary Figure S1). In both sexes, treatment with BAPN blocked the increase in the stiffness variability index of the subendothelial matrix induced by chronic d-flow (Table 2).

We conclude that chronic exposure to d-flow significantly increases endothelial and subendothelial stiffness in saline-treated mice, an effect largely prevented by BAPN treatment.

### The mechanical properties and morphology of endothelial cells depend on substrate stiffness

**FIGURE 4:**
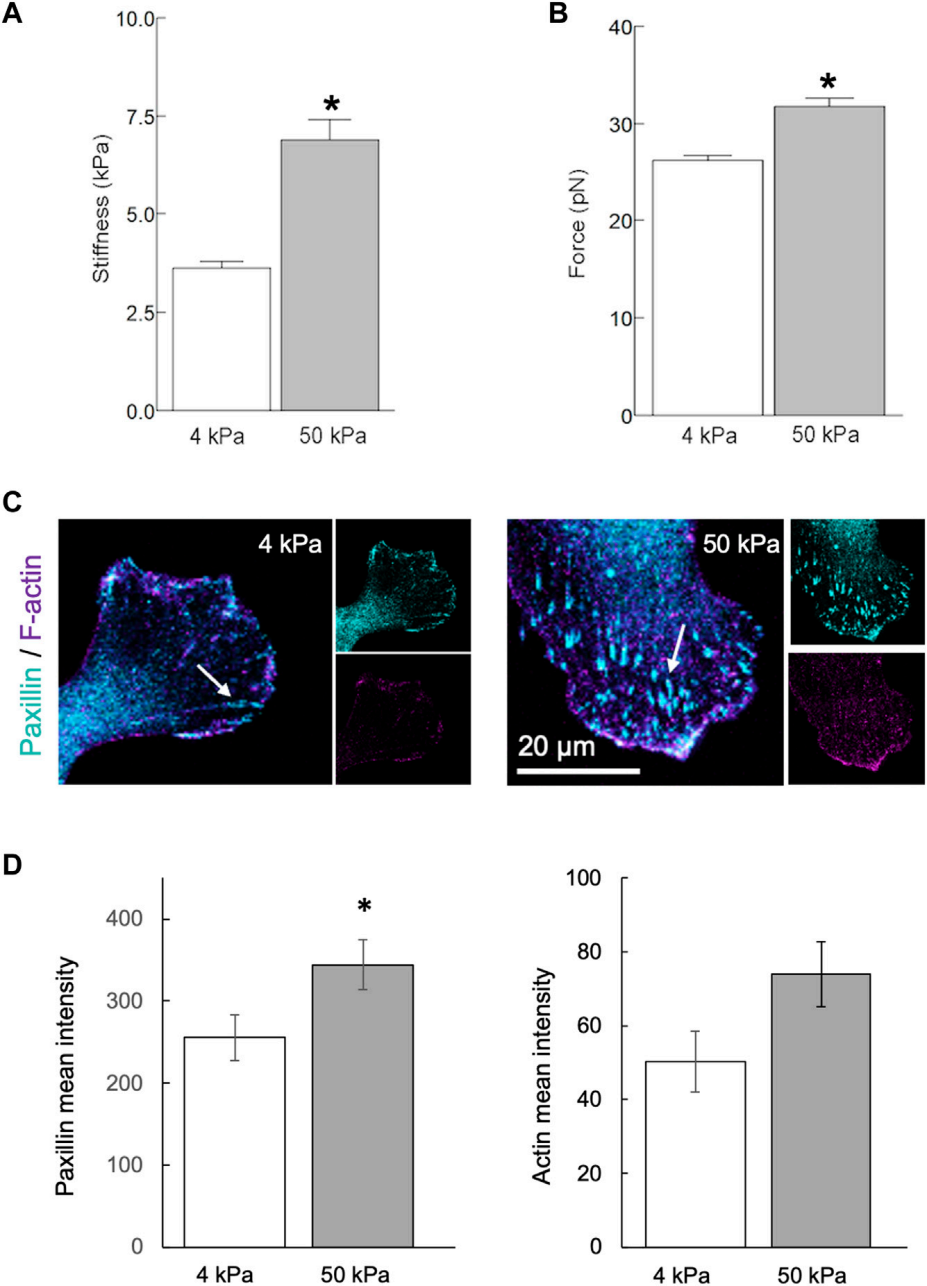
Matrix stiffness modulates the endothelial phenotype *in vitro*. Human aortic endothelial cells were cultured on polyacrylamide hydrogels with stiffnesses of 4 or 50 kPa for 24 h. **(A,B)** AFM measurements using a fibronectin-coated pyramidal tip enabled acquisition of integrin-to-fibronectin adhesion forces and cells stiffness at the point of contact. Data summarizing three independent experiments including a minimum of 10 cells examined per experiment. The number of force curves acquired for each condition is indicated in parentheses as follows: 4 kPa (1344), 50 kPa (1330). **(C,D)** Recruitment of paxillin at focal adhesions [arrows, **(C)**] was significantly increased (*p* < 0.05) in endothelial cells cultured on stiff (4 kPa) vs. soft matrices (50 kPa). A similar trend was also observed in the organization of actin cytoskeleton **(D)**. Data shown as mean ± sem. * *p* < 0.05.

To determine the effect of substrate stiffness on human aortic endothelial cells (HAEC) *in vitro*, cells were seeded on collagen-coated polyacrylamide hydrogels. Soft (4 kPa) and stiff (50 kPa) hydrogels were used to mimic, respectively, the mechanical properties of the healthy and diseased arterial wall. Twenty-four hours after seeding, stiffness and ⍺5β1 integrin-fibronectin adhesion force at the point of contact were measured in live cells. HAEC cultured on stiff hydrogels were stiffer (*p* < 0.05) than those cultured on soft hydrogels (Figure 4A). Cells cultured on stiffer hydrogels also exhibited increased stiffness variability, as reflected by the variability index (4 kPa: 3.0; 50 kPa: 5.6). In addition, integrin adhesion force was higher (*p* < 0.05) in cells cultured on stiff vs. soft hydrogels (Figure 4B). Consistent with this data, the development of focal adhesions, revealed by the clustering of the focal adhesion-associated protein paxillin, was significantly increased (*p* < 0.05) in endothelial cells cultured on stiff vs. soft matrices (Figures 4C, D). A similar trend was also observed in the organization of the actin cytoskeleton (Figures 4C, D).

In conclusion, endothelial cells cultured on stiffer substrates exhibit increased cell stiffness, greater integrin adhesion force, enhanced focal adhesion formation, and more organized actin cytoskeleton compared to those cultured on softer substrates.

## Discussion

A major finding of this study is that pharmacological inhibition of collagen crosslinking by targeting LOX enzymes ameliorates intimal stiffening induced by chronic exposure to d-flow. Of note, these effects were consistently observed in arteries from male and female mice. Thickening and stiffening of the intima in areas where the arterial tree undergoes geometric transitions (such as bifurcations, branch vessels, and curvatures) may constitute an initially adaptive response to changes in local hemodynamics. However, when combined with additional stressors such as metabolic imbalance, hypertension, or aging, altered blood flow patterns in these areas can lead to the formation of atherosclerotic plaque [36, 37]. Therefore, identifying targets that are uniquely sensitive to arterial wall stiffening induced by persistent d-flow, in the absence of confounding atherogenic factors, could potentially advance the development of new therapeutic approaches. These new strategies would complement existing cholesterol-lowering therapies for arterial disease.

Measurements performed just 2 days after PCL provided insights into the effects of acute exposure to d-flow in the absence of changes in the stiffness of the subendothelial matrix. Overall, our results suggest that a) acute, short-duration exposure to disturbed flow *in vivo* has limited effects on the mechanical phenotype of the endothelium and sub-endothelial matrix, b) there is a consistent difference in endothelial stiffness between females and males, regardless of the flow regime, even in the absence of differences in sub-endothelial matrix properties, and c) irrespective of sex or flow regime, the sub-endothelial matrix is consistently stiffer than the endothelium.

Unexpectedly, the endothelium was softer in acutely ligated arteries of males. Acute exposure to oscillatory shear stress was shown to induce transient endothelial dysfunction, assessed by sustained stimulus flow-mediated dilation, in men but not in women [38]. Studies *in vitro* have also identified sex-dependent responses of endothelial cells exposed to combinations of shear stress and substrate stiffness; overall endothelial cells from males were more sensitive than those from females to changes in the mechanical microenvironment [39]. Further studies coupling analysis of mechanical and functional properties of the arterial wall will eventually elucidate the significance of sex-differences in the endothelial response to short-term disturbed blood flow *in vivo*.

Enzymes of the LOX family play a key role in collagen and elastin crosslinking, thereby contributing to their mechanical properties, i.e., tensile strength and elasticity. The LOX family of enzymes catalyze the oxidative deamination of lysine and hydroxylysine residues to generate highly reactive lysyl-aldehyde (allysine) which, in turn, undergoes spontaneous reactions with other lysine, hydroxylysine or allysine residues to create crosslinks. Previous research showed that a feed-forward loop involving extracellular matrix deposition and LOX-dependent collagen crosslinking promotes atherosclerosis progression in hyperlipidemic, ApoE-deficient mice [40]. In such context, arterial wall softening by the LOX family inhibitor BAPN attenuated atherosclerotic plaque formation. Here, we combined 2 weeks of PCL with continuous delivery of BAPN in wild type (C57BL/6J) mice fed regular chow. Our study, therefore, examined the direct contribution of LOX activity to arterial wall stiffening induced by chronic d-flow without confounding effects of hypercholesterolemia or ApoE inactivation. In line with previous findings [40], our results showing prevention of d-flow-induced intimal stiffening by BAPN administration suggests that extracellular matrix-targeted approaches that preserve or restore the mechanical homeostasis of the arterial wall may prove effective in reducing the burden of cardiovascular disease.

Alterations in the mechanical characteristics of the arterial wall can potentially influence the communication between endothelial cells and smooth muscle cells. For example, compared to the healthy endothelium of compliant arteries in young mice, the compromised nitric oxide release by the dysfunctional endothelium of stiff arteries in aged mice results in increased synthesis and secretion of LOX enzymes by smooth muscle cells [6]. These observations align with those of Jo and colleagues [12] who used the PCL model to demonstrate that persistent but not short-term exposure to d-flow impairs endothelium-dependent, nitric oxide-mediated arterial relaxation. The present observations, in conjunction with those of others [12, 17], suggest the existence of a mechanically driven feedback loop. This loop entails the disruption of endothelial homeostasis by prolonged exposure to d-flow, which fosters stiffening of the arterial wall. This, in turn, exacerbates endothelial dysfunction. Significantly, links between endothelial stiffening and various indicators of endothelial dysfunction, including decreased release of nitric oxide, increased permeability [41, 42], elevated expression of cell adhesion molecules [43], and augmented leukocyte adhesion [44], have been well documented. In agreement with previous studies [42, 45, 46], our results from *in vitro* experiments show that higher substrate stiffness strengthens focal adhesion formation and binding to the matrix, which in turn, increases the organization of the actin cytoskeleton and cell stiffness. Endothelial cell stiffening, resulting from increased actin reorganization, was shown to promote a pro-inflammatory state linked to increased ICAM-1-mediated leukocyte trans-endothelial migration [43].

The process of elastic fiber formation spans embryonic development and ceases in early postnatal life [47]. Stiffening and remodeling of mature arteries, on the other hand, are accompanied by elastin fragmentation and increased collagen deposition. Given that the activity of enzymes of the LOX family is primarily directed towards the newly deposited collagen, particularly in response to d-flow, we suggest that the observed softer nature of the sub-endothelial matrix in LCA + BAPN compared to control RCA + saline arteries likely results from reduced collagen crosslinking. This is supported by the presence of more prominent collagen fibers in ligated arteries treated with saline vs. those treated with BAPN, as shown by SHG imaging. In addition, BAPN restored, to a significant extent, the mechanical homeostasis of the endothelium. Nonetheless, we observed that the endothelium of ligated, BAPN-treated carotids was ∼2-fold stiffer than that of unligated, saline treated counterparts. Thus, in the absence of subendothelial matrix stiffening, chronic stress from d-flow may be sufficient to induce subtle changes in the mechanical phenotype of the endothelium. This mechanism is supported by recent work suggesting that endothelial stiffening may occur without changes in the stiffness of the arterial wall [48] or the subendothelial matrix [25, 49]. Of clinical significance, Levitan and colleagues demonstrated that endothelial stiffening and dysfunction, arising from the combined effects of d-flow and dyslipidemia or aging, leads to early stages of atherogenesis.

While the deliberate inclusion of sex as a biological variable of importance in cardiovascular research has increased in recent years, much of the literature is built on studies excluding females or presenting results combining observations from males and females without rigorous testing for sex specific effects [50, 51]. Our study shows comparable intimal stiffening in arteries from male and female mice exposed to persistent d-flow, an effect that was substantially mitigated by BAPN treatment in both sexes.

Pharmacological inhibition of LOX enzymes to reduce collagen crosslinking is well-supported by multiple lines of evidence as follows: *i*) wild-type mice subjected to partial carotid ligation (PCL) exhibit increased collagen deposition and arterial wall stiffening [17], *ii*) a rat model of intimal hyperplasia demonstrates elevated LOX expression [22], *iii*) hypercholesterolemic, ApoE-deficient mice [40], as well as aged wild-type mice [6], exhibit increased LOX activity associated with arterial stiffening, and *iv*) various preclinical models of hypertension show LOX up-regulation linked to enhanced oxidative stress and vascular stiffness [23]. Furthermore, low shear stress induces increased LOX expression in endothelial and vascular smooth muscle cells [24]. The collective evidence highlights the central role of LOX family activity as a prominent target for addressing arterial stiffening across diverse etiologies. It is worth noting that administration of BAPN, an irreversible inhibitor of enzymes of the LOX family [52], does not cause aortic pathology when administered alone in adult mice [53].

The present investigation underscores the potential of inhibiting collagen crosslinking by LOX enzymes as an effective strategy to offset intimal stiffening. Future studies examining changes in molecular profiles and cellular phenotypes linked to persistent d-flow, coupled with strategies aimed at preserving or restoring the mechanical homeostasis of the arterial wall, hold the potential to unveil novel targeted interventions aimed at promoting arterial health.

## Conclusion

This study was primarily designed to test the hypothesis that chronic d-flow initiates a feed forward loop promoting arterial wall stiffening leading to altered endothelial mechanics; hence interventions aimed at reducing arterial stiffness have the potential to disrupt this loop and preserve the mechanical hemostasis of the endothelium. Whereas acute d-flow appears to exert minimal effects on the mechanical properties of the arterial intima, persistent d-flow leads to remarkable stiffening of both the endothelium and the subendothelial matrix. Intimal stiffening induced by persistent d-flow can be averted through pharmacological inhibition of collagen crosslinking by enzymes of the LOX family.

## Data Availability

The raw data supporting the conclusions of this article will be made available by the authors, without undue reservation.

## Supplementary material

SUPPLEMENTARY FIGURE S1 Second harmonic generation images of collagen in unligated (RCA) and ligated (LCA) carotid arteries obtained from male mice treated with saline or BAPN. The light grey traces represent collagen fibrils in the arterial wall. The images show more prominent collagen fibrils in the wall of LCA + saline compared to LCA + BAPN or unligated RCA arteries.
