# Supplementary figures and images for: Modulation of arterial intima stiffness by disturbed blood flow
Source: Exp Biol Med (Maywood). 2024 Jul 31;249:10090. doi: 10.3389/ebm.2024.10090 (PMC11323813; doi:10.3389/ebm.2024.10090)

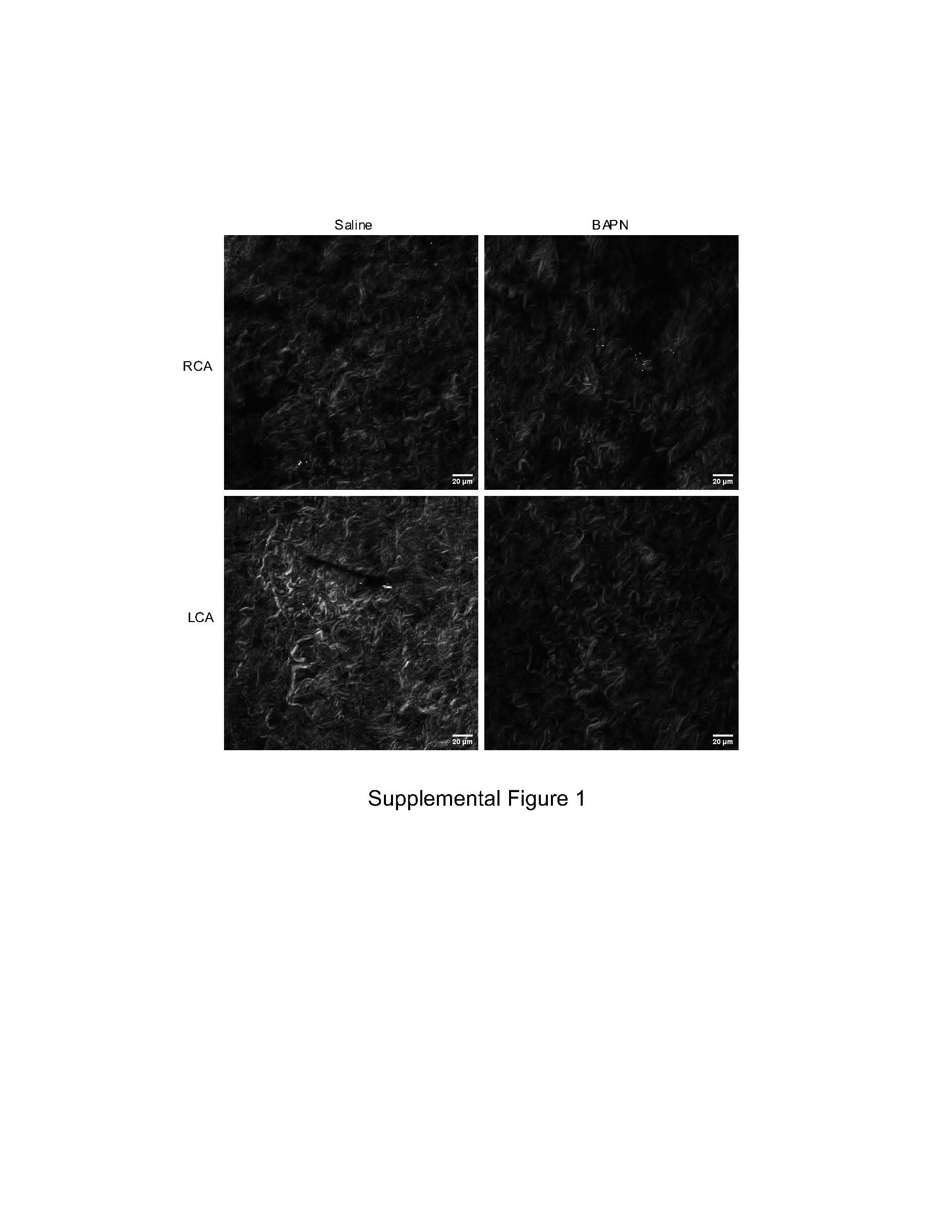

Supplement: Supplementary file 1 [file Image1.jpeg]
